# Model-based assessment of mammalian cell metabolic functionalities using omics data

**DOI:** 10.1016/j.crmeth.2021.100040

**Published:** 2021-06-30

**Authors:** Anne Richelle, Benjamin P. Kellman, Alexander T. Wenzel, Austin W.T. Chiang, Tyler Reagan, Jahir M. Gutierrez, Chintan Joshi, Shangzhong Li, Joanne K. Liu, Helen Masson, Jooyong Lee, Zerong Li, Laurent Heirendt, Christophe Trefois, Edwin F. Juarez, Tyler Bath, David Borland, Jill P. Mesirov, Kimberly Robasky, Nathan E. Lewis

**Affiliations:** 1 Novo Nordisk Foundation Center for Biosustainability at the University of California, San Diego, School of Medicine, La Jolla, CA 92093, USA; 2 Department of Pediatrics, University of California, San Diego, School of Medicine, La Jolla, CA 92093, USA; 3 Bioinformatics and Systems Biology Program, University of California, San Diego, La Jolla, CA 92093, USA; 4 Department of Medicine, University of California, San Diego, School of Medicine, La Jolla, CA 92093, USA; 5 Moores Cancer Center, University of California, San Diego, La Jolla, CA 92093, USA; 6 Department of Bioengineering, University of California, San Diego, La Jolla, CA 92093, USA; 7 Department of Computer Science and Engineering, University of California, San Diego, La Jolla, CA 92093, USA; 8 Luxembourg Centre for Systems Biomedicine, University of Luxembourg, Esch-sur-Alzette, Luxembourg; 9 Department of Biomedical Informatics, UC San Diego Health, University of California, San Diego, La Jolla, CA 92093, USA; 10 Renaissance Computing Institute, The University of North Carolina at Chapel Hill, Chapel Hill, NC 27517, USA; 11 Department of Genetics, University of North Carolina at Chapel Hill, Chapel Hill, NC 27514, USA; 12 School of Information and Library Science, University of North Carolina at Chapel Hill, Chapel Hill, NC 27599, USA; 13 Carolina Health and Informatics Program, University of North Carolina at Chapel Hill, Chapel Hill, NC 27599, USA

**Keywords:** metabolic function, omic data, systems biology, functional analysis, genome-scale metabolic network, transcriptomic

## Abstract

Omics experiments are ubiquitous in biological studies, leading to a deluge of data. However, it is still challenging to connect changes in these data to changes in cell functions because of complex interdependencies between genes, proteins, and metabolites. Here, we present a framework allowing researchers to infer how metabolic functions change on the basis of omics data. To enable this, we curated and standardized lists of metabolic tasks that mammalian cells can accomplish. Genome-scale metabolic networks were used to define gene sets associated with each metabolic task. We further developed a framework to overlay omics data on these sets and predict pathway usage for each metabolic task. We demonstrated how this approach can be used to quantify metabolic functions of diverse biological samples from the single cell to whole tissues and organs by using multiple transcriptomic datasets. To facilitate its adoption, we integrated the approach into GenePattern (www.genepattern.org—CellFie).

## Highlights

• An alternative approach for the interpretation of omics data is proposed
• A collection of tasks covering major metabolic activities in mammalian cells is presented
• The framework predicts how metabolic functions change on the basis of omics data
• Metabolic functions from single cells to tissues and organs can be quantified

## Motivation

The existence of complex interdependencies between genes, proteins, and metabolites challenge the interpretation of omics experiments. Data-driven approaches have been particularly useful for identifying gene sets of interest. However, it remains difficult to gain a mechanistic understanding of and to quantify a cell's functions from enriched ontology terms. Genome-scale systems biology models can be used to analyze these datasets, but they require specialized training and can take extensive effort to deploy. Here, we developed a framework to directly predict how changes in omics experiments correspond to cell or tissue functions, thereby facilitating phenotype-relevant interpretation of these complex datum types.

## Graphical abstract

**Figure:**
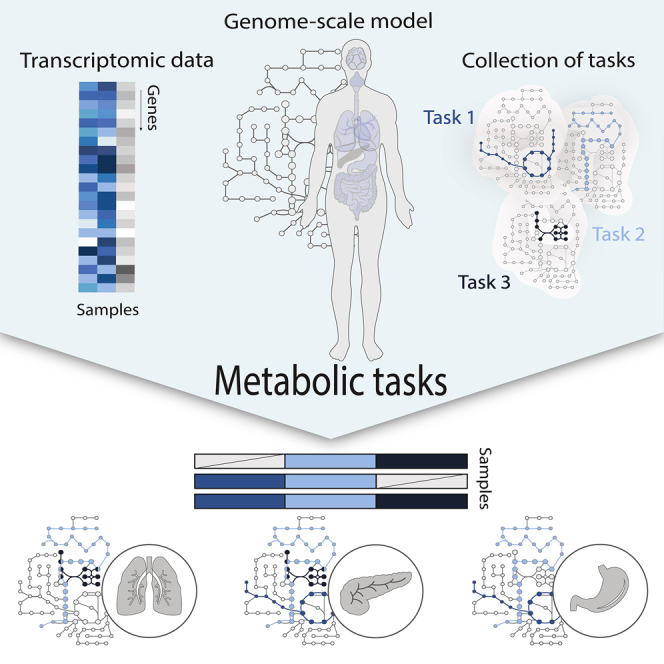

## Teaser

Richelle et al. present a framework to comprehensively quantify the propensity of a mammalian cell to be responsible for a metabolic function. This approach can be used to facilitate phenotype-relevant interpretation of transcriptomic datasets from the single-cell level of these biological entities to their organization in tissues and organs.

## Introduction

High-throughput omics technologies allow researchers to comprehensively monitor cells and tissues at the molecular level and record subtle molecular changes that might contribute to the acquisition of a specific phenotype. However, the complex interdependencies between the gene, protein, and metabolite components limit our capacity to identify the molecular basis of specific phenotypic changes. Therefore, it remains challenging to extract tangible biological meaning from omics data.

Many approaches exist to systematically interpret gene expression changes, ranging from simple enrichment analyses to detailed mechanistic systems biology modeling. Several user-friendly approaches have been developed that allow any researcher to test for enrichment in groups of genes, e.g., pathways, biological processes, or ontology terms (Huang et al., 2009; Khatri et al., 2012). Such approaches are invaluable for identifying groups of genes that are more frequently differentially expressed, but the methods are limited in their capacity to describe how the differential changes affect cellular metabolic functions. To interpret the impact on function, mathematical models of pathways can be used. For example, genome-scale metabolic network reconstructions are knowledge bases of all metabolic pathways in an organism (Feist et al., 2009; Gu et al., 2019; Robinson et al., 2020). These networks directly link genotype to phenotype, given that they mathematically describe the mechanisms by which all cell parts (e.g., membranes, proteins) are concurrently made. Thus, approaches have emerged to analyze omics data in the context of these models (Blazier and Papin, 2012; Lewis et al., 2009), yielding a wealth of detailed insights into the mechanisms underlying complex biological processes (Bordbar et al., 2014). However, these approaches are not widely used because they are quite complex, requiring months of analysis by experts with years of specialized training.

Here, we propose an alternative approach for the interpretation of omics data (e.g., differentially expressed genes) that captures the simplicity of enrichment analyses while providing mechanistic insights into how differential expression affects specific cellular functions, based on pre-computed model simulations. To this end, genome-scale metabolic networks were decomposed into many smaller metabolic tasks (Blais et al., 2017; Thiele et al., 2013). We curated and standardized these tasks, resulting in a collection of hundreds of tasks covering seven major metabolic activities of a cell (energy generation, nucleotide, carbohydrate, amino acid, lipid, vitamin and cofactor, and glycan metabolism). We further developed a framework to directly predict the activity of these metabolic functions from transcriptomic data. To this end, we used genome-scale models of mammalian metabolism to define gene sets responsible for the activation of pathways required for each specific metabolic task. Through this platform, users can overlay their data and comprehensively quantify the propensity of a cell line or tissue to be responsible for a metabolic function. Finally, we demonstrate the capacity of this approach to leverage metabolic functions of human cells and tissues by using transcriptomic data from the Human Protein Atlas (Uhlén et al., 2015) and show how the identification of metabolic tasks can be used to understand the organization of these biological entities into broader functional organ systems. Furthermore, using data from the Single-Cell Atlas of Adult Mouse Brain (Saunders et al., 2018), we show cell type specificity of several metabolic functions. Finally, we highlight the potential applications of this method to drive the discovery of new drug targets by identifying the main metabolic dysregulations associated with Alzheimer’s disease by using single-cell transcriptomic data from the ROSMAP (Religious Orders Study and Memory Aging Project) dataset (Bennett et al., 2018).

## Results

### A framework to quantify a cell's metabolic functions

Cells deploy diverse molecular functions to interface with their microenvironment and adapt these as needed to cope with environmental changes. In metabolism, small modules of reactions can be defined as metabolic tasks (i.e., the generation of specific product metabolites given a defined set of substrate metabolites). The library of metabolic tasks a cell can sustain is embedded in its genome, and the capacity to modulate the activity of these tasks enables the cell's adaptation to a changing environment.

**Figure 1:**
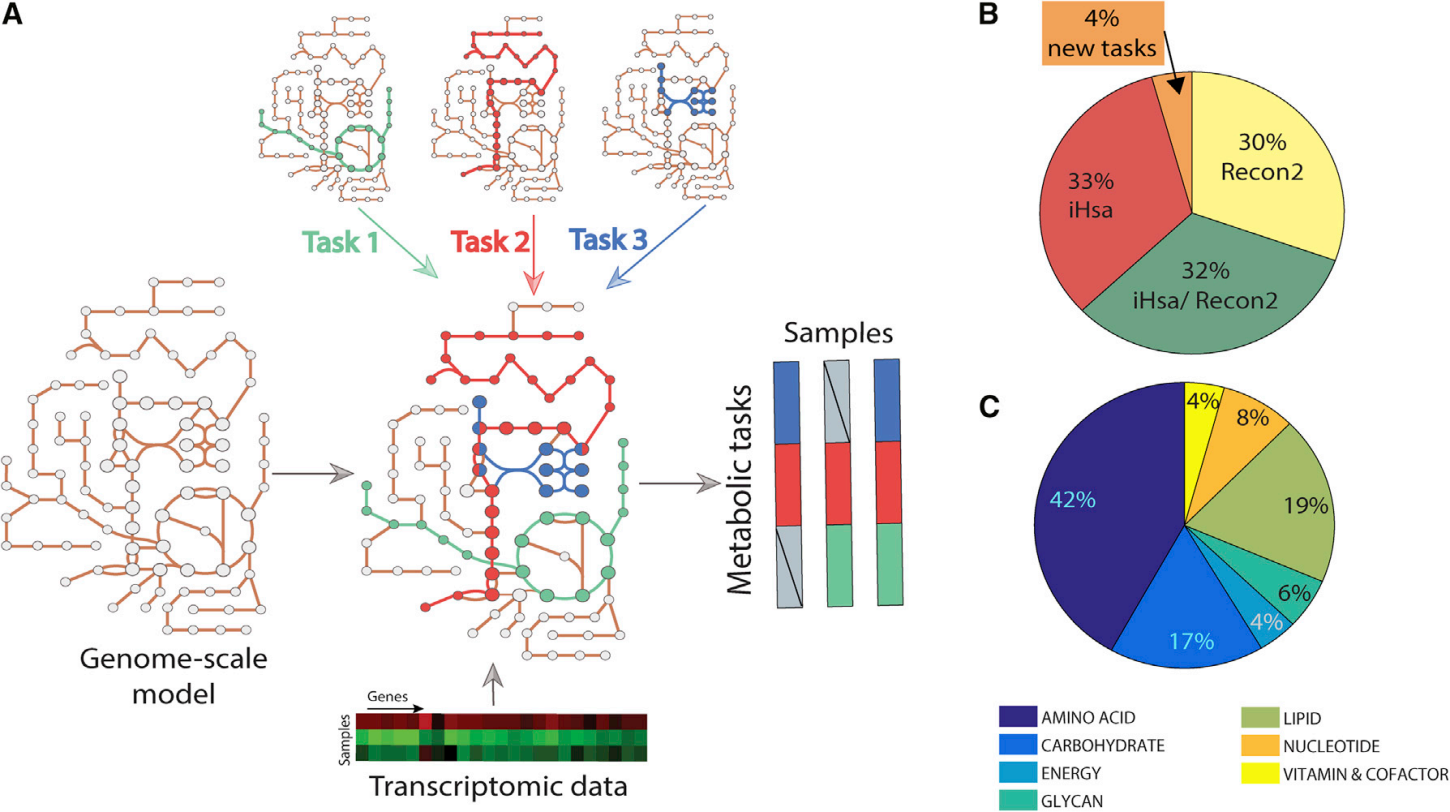
Genome-scale metabolic models can be used to infer the activity of a defined list of metabolic functions (A) Metabolic tasks are a modeling concept that we extend here to infer metabolic functions from transcriptomic data. (B) We curated and reconciled a collection of 195 tasks, derived in large part from earlier modeling studies (i.e., Recon2 and iHsa). The original source of each task and comments on the biological evidence of the associated metabolic function are presented in Table S1. (C) The list of curated tasks covers seven main metabolic systems.

This concept of “metabolic tasks” has been previously used to evaluate the quality and capabilities of genome-scale metabolic models (Duarte et al., 2007; Thiele et al., 2013; Blais et al., 2017; Gille et al., 2010; Mardinoglu et al., 2014; Uhlén et al., 2015; Agren et al., 2014; Bordbar et al., 2012). However, these studies used various frameworks to define the cell's capacity to sustain a metabolic task (as described previously [Richelle et al., 2019a]). Therefore, the library of metabolic tasks differed across studies in content and form, preventing the comparison of results from the various studies. Thus, we first manually collated, curated, and standardized existing metabolic task lists (Blais et al., 2017; Thiele et al., 2013), resulting in a documented collection of 195 tasks covering seven major metabolic activities of a cell (energy generation, nucleotide, carbohydrates, amino acid, lipid, vitamin and cofactor, and glycan metabolism) (Figure 1 and Table S1). We further unified the formalism of the metabolic tasks and the associated computational framework for their use in the modeling context (detailed in our earlier study [Richelle et al., 2019a]).

Here, we extend this concept beyond model benchmarking by developing a platform that quantifies a cell's metabolic functions directly from transcriptomic data. To achieve this, we used genome-scale metabolic models to identify the list of reactions required to accomplish each metabolic task and to identify the list of genes that might contribute to the acquisition of this metabolic function on the basis of Gene Protein Reaction (GPR) rules. With only 195 tasks, we can capture the activity of 40% of the metabolic genes in the human genome-scale networks (43.94% for Recon2.2 [Swainston et al., 2016] and 37.36% for iHsa [Blais et al., 2017]).

The proposed computation of the metabolic score (i.e., relative activity of a metabolic task) relies first on the preprocessing of the available transcriptomic data and the attribution of a gene activity score for each gene (Richelle et al., 2019b). We further selected the genes responsible for the activation of each reaction required for a task by using the GPR rules and average their activity to compute the metabolic task score (see STAR Methods for more details). In doing so, transcriptomic data can be directly used to quantify the relative activity of each metabolic function in a specific condition. Importantly, given that gene lists are pre-computed, no modeling background is required for the user.

### Metabolic tasks can leverage metabolic functions of human tissues

**Figure 2:**
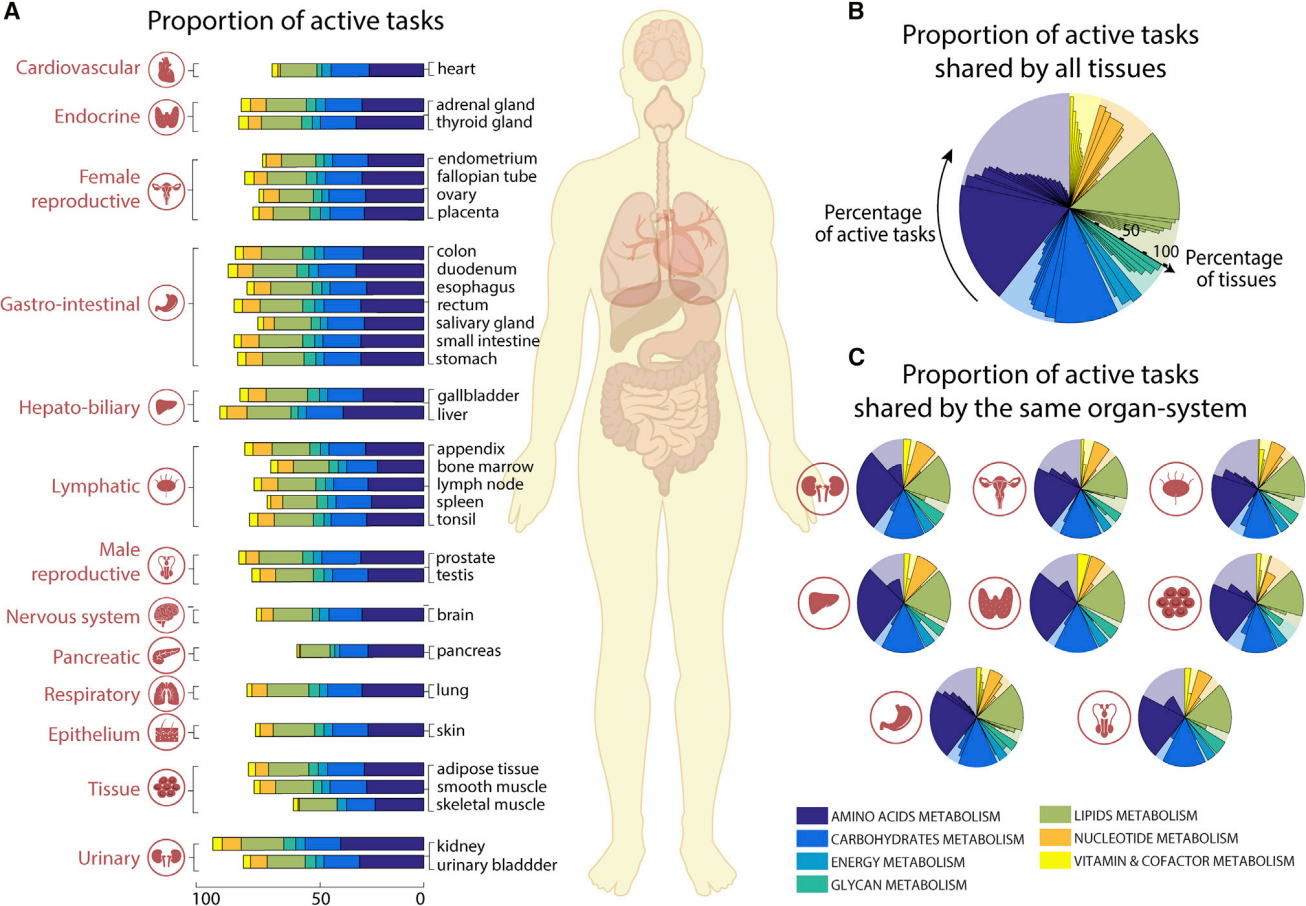
Metabolic tasks capture functional similarities between human tissues (A) The proportion of tasks identified as active in the seven major metabolic activities for each of the 32 tissues present in the Human Protein Atlas (Uhlén et al., 2015). (B and C) Shown are (B) the percentage of active tasks that are shared by all tissues and (C) those shared within the same organ systems (Table S3). The background shaded color distribution represents the assignment of the 195 curated tasks to seven main metabolic systems.

Each organ, tissue, and cell type in the human body has a distinct set of specific functions. The functions of each cell type are integrated to achieve the functions of each tissue, organ, and organ system. Because there is no central database comprehensively describing the unique metabolic functions of different tissues, we used transcriptomic data from the Human Protein Atlas (Uhlén et al., 2015) to quantify the metabolic functions of 32 tissues by using Recon2.2 (Swainston et al., 2016) as reference genome-scale model (Figure 2A; Tables S2 and S3). We observed that >40% of the tasks are shared by all tissues (i.e., 79 tasks, Figure 2B), and within organ systems even more tasks were shared (Figure 2C and Table S3). To assess the significance of this common set of tasks, we collected a list of known housekeeping genes (Blomen et al., 2015; Eisenberg and Levanon, 2013; Hart et al., 2017; Wang et al., 2015). This list included 411 metabolic genes from Recon2.2 (Swainston et al., 2016) (24.5% of all metabolic genes in Recon2.2). Interestingly, we found that 97.5% of tasks shared by all the tissues (i.e., 79 tasks, Figure 2B) are associated with at least one housekeeping gene. This included 277 housekeeping genes covered by metabolic tasks, which represent 67.4% of all Recon2.2 housekeeping genes.

### Metabolic tasks successfully cluster histologically similar tissues

**Figure 3:**
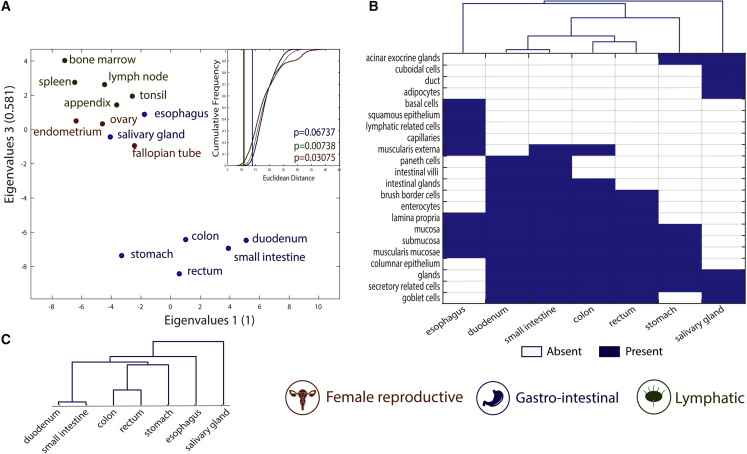
Metabolic tasks capture the histological similarities of tissues (A) Visual representation of the similarity between tissues computed on the basis of the metabolic task approach using a principal coordinates analysis. The mean Euclidean distance for 100,000 randomly selected groups with the same number of tissues (inset) highlights the significance of the tissues clustering into organ systems. The vertical lines are the mean Euclidean distance between tissues belonging to the same organ system and their empirical p value (see STAR Methods for more details). (B) Heatmap and hierarchical clustering of histological similarities between tissues of the gastrointestinal group. (C) Hierarchical clustering of similarities between tissues of the gastrointestinal group computed on the basis of the metabolic task approach.

We further analyzed the similarities of metabolic tasks of tissues within the same organ systems as classified in the Human Protein Atlas (Uhlén et al., 2015). Specifically, we compared the similarities of tissues belonging to three different organ systems (i.e., female reproductive system, gastrointestinal tract, and lymphatic system; see STAR Methods for more details). We found that the metabolic task approach successfully groups tissues by organ system (Figures 3A and S1 show the clusters from the binary version of the metabolic task approach).

The gastrointestinal system presents the lowest grouping significance, as two tissues seem to be group outliers (i.e., esophagus and salivary gland). Interestingly, these two tissues are histologically substantially different from the rest of the gastrointestinal system. Specifically, they are the only tissues without columnar epithelium. The salivary gland is the only tissue in this group having cuboidal cells in its epithelium, whereas the esophagus contains squamous epithelium (Figure 3B). The histological distance between tissues belonging to the gastrointestinal system was successfully captured by metabolic task analysis (Figure 3C).

### Metabolic task analysis captures tissue- and cell-specific functions

**Figure 4:**
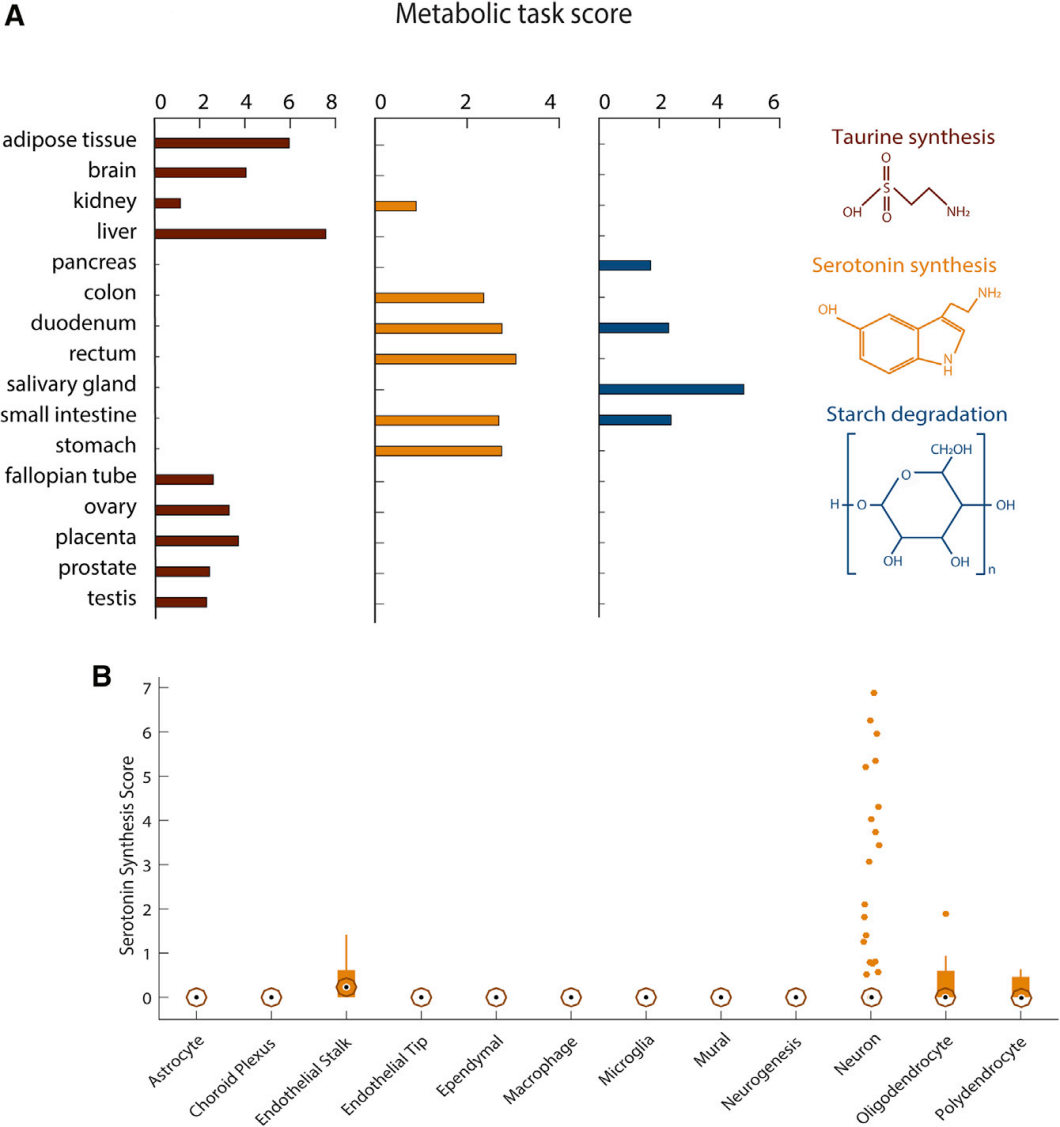
Metabolic specificities of tissues and brain cells (A) Metabolic task scores associated with the synthesis of taurine and serotonin and the degradation of starch. Note that the figure presents only the 16 tissues for which these tasks have been predicted. (B) Score associated with the synthesis of serotonin for 12 different brain cell types. The central black mark indicates the median, and the bottom and top edges of the box indicate the 25th and 75th percentiles, respectively. The whiskers extend to the most extreme data points not considered outliers, and the outliers are plotted individually by using orange circles with dots.

Some metabolic functions only occur in specific organs, tissues, or cells. For example, taurine is the major constituent of bile secreted by the liver, and its biosynthesis also occurs in the kidney and brain (Ripps and Shen, 2012). Furthermore, taurine plays an important role in maintaining normal reproductive functions of mammals (Lobo et al., 2000; Mu et al., 2015). Metabolic task analysis shows taurine synthesis in those known tissues and reproductive tissues (Figure 4A). Similarly, metabolic task analysis predicts that starch degradation occurs in the digestive tissues, consistent with the reported localization (Ao et al., 2007). Thus, the analysis can capture tissue-specific metabolism.

Serotonin biosynthesis is similarly accurately predicted to be synthesized in the gastrointestinal tract. However, the method does not predict its known synthesis by the brain (Berger et al., 2009). This can be expected, as serotonergic neurons are localized to the raphe nuclei, whereas the bulk brain transcriptomic data in the Human Protein Atlas RNA sequencing (RNA-seq) were sampled from cerebral cortex (Uhlén et al., 2015). Thus, we used the metabolic task approach on single-cell RNA-seq data of the adult mouse brain (Saunders et al., 2018) (Tables S5 and S6) and found that serotonergic neurons can be successfully identified (Figure 4B).

### Metabolic task analysis captures the differences between brain cell types

**Figure 5:**
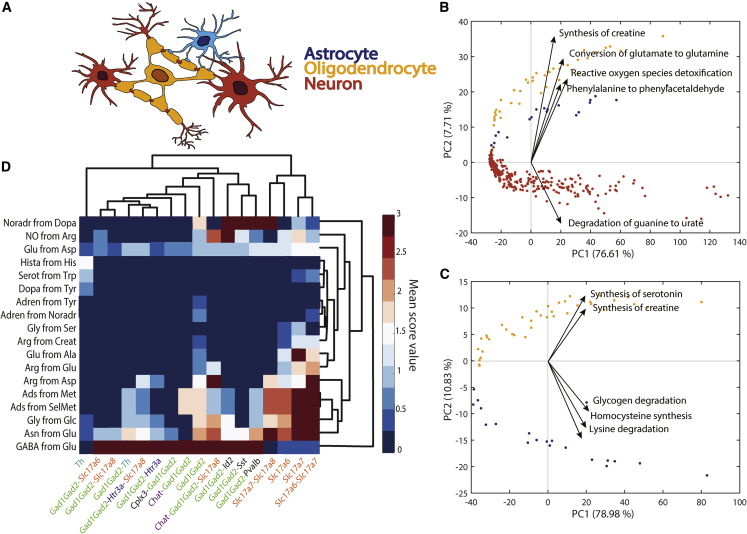
Metabolic differences between astrocytes, neurons, and oligodendrocytes (A) Schematic representation of spatial connection between astrocytes (blue), neurons (red), and oligodendrocytes (yellow). (B) Principal component analysis (PCA) component scores for the three different cell types (astrocytes, blue; neurons, red; oligodendrocytes, yellow) and the five dominant tasks in the second principal component. The five tasks most influencing the third principal component are presented in Figure S2A. (C) PCA component scores for only two cell types (astrocytes, blue; oligodendrocytes, yellow) and the five dominant tasks in the second principal component. The five tasks most influencing the third principal component are presented in Figure S2B. (D) Heatmap of metabolic tasks score mean values associated with the synthesis of main neurotransmitters in the context of the gene markers for different neuron types (i.e., mean of the metabolic task score obtained for all samples associated with specific set of gene markers). The known gene markers are highlighted with different colors (e.g., GAD family in green, Slc17 gene family in orange, Chat gene marker in purple).

The human brain is a metabolically demanding organ consisting of diverse cell types, each one with unique metabolic capabilities. Although some metabolic interchanges between brain cell types are well known (e.g., glutamate-glutamine shuttle between neurons and astrocytes), there remain many open questions concerning the specific contribution of each cell type in brain function. Thus, we used single-cell RNA-seq data from adult mouse brain (Saunders et al., 2018) to assess the main metabolic features that differentiate astrocytes, neurons, and oligodendrocytes (Figure 5A; see STAR Methods for details). The metabolic task approach clearly differentiates the three cell types and details their metabolic specialization (Figures 5B, 5C, S2, and S3). Our analysis confirms previously known specific metabolic features such as the evidence that astrocytes fuel the glutamate-glutamine shuttle (Amaral et al., 2013) (Figure 5B) and that oligodendrocytes are likely the primary source of creatine in the brain (Chamberlain et al., 2017) (Figure 5C). Interestingly, there has been a debate as to whether oligodendrocytes serve as sources of glutamine synthesis (Anlauf and Derouiche, 2013) in the glutamate-glutamine shuttle. Our analysis of single-cell RNA-seq clearly supports this hypothesis (Figures 5B and S3D).

To analyze the capacity of this method to be used to resolve open questions, we also created a new set of tasks specific to neurotransmitter synthesis (Table S6). We compared the expression of these tasks with respect to the type of gene markers used to differentiate the single cells. We observe that each set of gene markers used for identifying the different clusters of neurons in the Single-Cell Atlas of Adult Mouse Brain (Saunders et al., 2018) are associated with specific neurotransmitter patterns (Figure 5D). Specifically, the Slc17 gene family is associated with the non-expression of the GABA neurotransmitter presumably corresponding to glutamatergic neurons. Contrarily, all the neurons identified by using GAD family gene markers are associated with a high GABA synthesis corresponding to GABAergic neurons (Saunders et al., 2018). Interestingly, tyrosine hydroxylase is a marker of dopaminergic neurons (Contini et al., 2010), and we observe that the neurons identified with this gene are the only ones presenting the synthesis of dopamine.

### Metabolic task analysis highlights metabolic dysregulations in Alzheimer's disease

**Figure 6:**
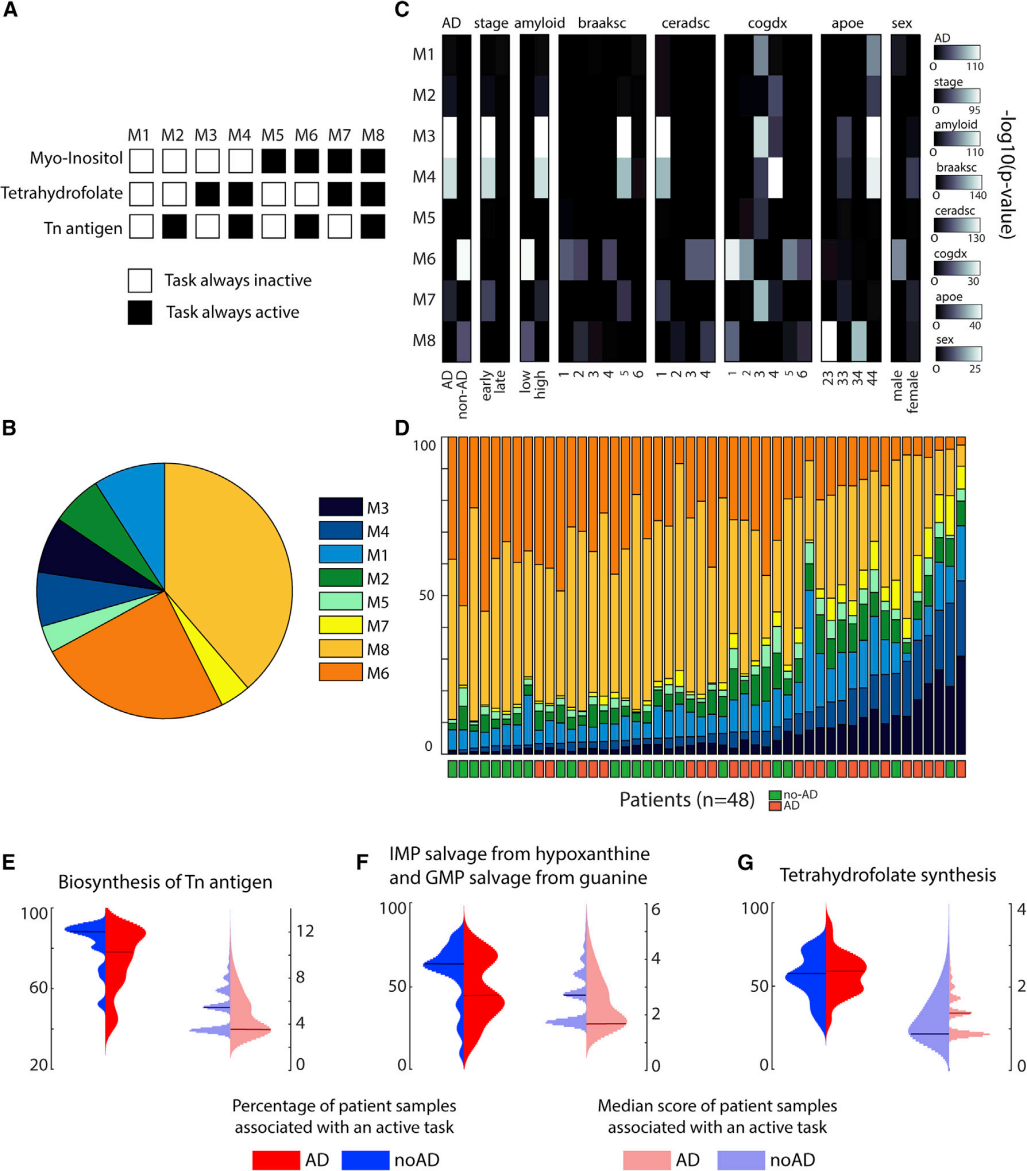
Metabolic clusters of excitatory neurons and their link with Alzheimer's disease (A) The single-cell transcriptomic dataset was clustered into eight metabolic clusters with distinct patterns of activity for three metabolic tasks. (B) Percentage of the representation of each metabolic cluster within the ROSMAP dataset (Mathys et al., 2019). (C) Enrichment analysis (one-tailed Fisher's exact test) within each metabolic cluster of clinic-pathological variables (Mathys et al., 2019) (AD, pathology; stage, stage of the disease; amyloid, overall amyloid level; braaksc, Braak stage; ceradsc, assessment of neuritic plaques; cogdx, clinical consensus diagnosis; apoe, APOE (apolipoprotein E) genotype; sex, sex of the patient). (D) Percentage of samples of each metabolic cluster from each patient and their associated Alzheimer's diagnosis. (E–G) Expression patterns of the metabolic tasks (left: percentage of patient samples associated with an active task; right: related median score) presenting a dysregulated activity across groups of patients with different diagnosis for Alzheimer's disease (blue and red represent patients without and with Alzheimer's disease, respectively). The horizontal lines represent the median of the distribution.

Alzheimer's disease is a neurodegenerative disorder affecting millions of people, but to date we lack a cure. Despite decades of research into the disease, many questions remain regarding the molecular basis of its progression. However, increasing evidence suggests that metabolic dysfunction might contribute to nervous system degeneration (Butterfield and Halliwell, 2019; Kang et al., 2017; Lewis et al., 2010b). Whether metabolic alterations are the cause or the consequence of the pathogenesis remains unclear, but metabolic pathways might themselves contain potential targets for future therapies (Cai et al., 2012). In this context, we used single-cell RNA-seq data from ROSMAP (Bennett et al., 2018) to elucidate the main metabolic dysregulations associated with Alzheimer's disease. To this end, we clustered the excitatory neuron samples and identified the tasks that were active in more than 50% of the dataset. Only three metabolic tasks correspond to this criterion: the conversion of phosphatidyl-1D-*myo*-inositol to 1D-*myo*-inositol 1-phosphate, the synthesis of tetrahydrofolate, and the synthesis of “Tn antigen” (i.e., glycoprotein *N*-acetyl-D-galactosamine). We further used them to divide the samples into eight metabolic clusters depending on the combination of their activity in each sample (Figures 6A and 6B; see STAR Methods for more details). For each metabolic cluster, we tested their associations with pathological traits by using a one-tailed Fisher’s test (Figure 6C) and observed that specific metabolic clusters were enriched in samples associated with either Alzheimer's pathology (clusters M3 and M4) or no pathology (cluster M6). Interestingly, we were able to group the 48 patients from the dataset depending on their disease prognosis with 75% accuracy by sorting them with respect to the proportion of their samples in M3 and M4 (Figure 6D). Note that we applied the clustering approach and subsequent trait enrichment analysis to the six major cell types identified in the original study presenting this dataset (Mathys et al., 2019), and we did not find such a strong correlation for the other brain cell types (Table S7).

To better understand the metabolic functions differentiating the eight clusters, we computed the median of the combined metabolic task score (i.e., score in its binary version multiplied by the continuous one) and observed that only 13 tasks presented a median score different from zero in a metabolic cluster. We further used these identified tasks to investigate their expression patterns (i.e., percentage of patient samples associated with an active task and related median score) across the groups of patients presenting or not presenting a positive diagnosis for Alzheimer's disease. We observed distinct median score distributions depending on diagnosis for four tasks previously highlighted in the literature as being implicated in the Alzheimer's disease (Figures 6E–6G): the synthesis of Tn antigen (Frenkel-Pinter et al., 2017; Schedin-Weiss et al., 2014) (glycoprotein *N*-acetylgalactosamine), the synthesis of tetrahydrofolate (Troesch et al., 2016), and the salvage of inosine 5′-monophosphate and guanosine 5′-monophosphate (Garcia-Gil et al., 2018). Although the other metabolic tasks identified do not present distinct patterns at the level of the median score distribution, we observe that healthy subjects often present a higher percentage of samples for which these tasks are active (Figure S4). Thus, an overall deficiency of these metabolic activities is observed in patients with Alzheimer's disease. Interestingly, some dysregulated metabolic tasks have been observed in previous studies, such as pyridoxal phosphate synthesis (di Salvo et al., 2012), the presence of the thioredoxin synthesis (Silva-Adaya et al., 2014), fructose degradation (Cisternas et al., 2015), and the conversion of *myo*-inositol (Chhetri, 2019), whereas the others have not been specifically investigated. In this context, the metabolic dysregulations identified with our approach provide a hypothesis of new potential drug targets.

## Discussion

Here, we present an approach to predict the activity of hundreds of metabolic functions from transcriptomic data. This framework enables the comprehensive quantification of the propensity of a cell line or tissue to express a metabolic function, thereby facilitating phenotype-relevant interpretation of these complex datum types. We used multiple omics datasets to highlight the power of our approach to quantify metabolic functions from organ systems to single cells.

Enrichment analyses are invaluable for identifying gene classes that are significantly over- or under-represented in gene expression data. These gene groups can suggest functional biological processes by leveraging existing knowledge embedded in gene ontologies. Although these approaches are useful for genome-wide association studies and differential screening, they do not provide mechanistic details of metabolic pathway activities. Our framework, on the other hand, integrates omics datasets into pathways from computational models to quantitatively describe the genotype-phenotype relationship. The analysis of gene expression data with genome-scale systems biology models is well established and can provide deep mechanistic insights into the metabolic capabilities of a cell and/or tissue. Indeed, Uhlén et al. (2015) used a network-based approach and the concept of metabolic tasks to construct tissue-specific metabolic networks. The approach enforced the activity of tissue-specific metabolic tasks into each model to capture cellular functionalities known to occur in all cell types. In doing so, they also found metabolic housekeeping functions shared across all tissues and showed similarities between metabolic activities across tissues in the same organ systems. Unfortunately, the construction and analysis of such computational models is a complex and difficult task requiring expert knowledge of the tissues and modeling framework (Richelle et al., 2019a; Opdam et al., 2017). To overcome this problem, our framework successfully combines the capacity to provide mechanistic insights of network-based approaches and the simplicity of enrichment analyses. To further facilitate adoption of the approach, we integrated a CellFie module into the list of tools available in GenePattern (Reich et al., 2006) (www.genepattern.org; see STAR Methods for more details).

The list of metabolic functions presented in this study covers the functions of a substantial proportion of human metabolic genes (43.94% of the genes in Recon2.2 [Swainston et al., 2016] and 37.36% in iHsa [Blais et al., 2017]). Therefore, we focused here on demonstrating the use of the metabolic tasks rather than on the tasks themselves. However, this list can be easily expanded upon for mammalian cells and extended to diverse organisms and more cellular functions captured in systems biology models of metabolism, transcription, translation, and signaling. For example, genome-scale metabolic networks exist for hundreds of organisms, and updates on available networks are often released. A community standard for metabolic tasks will facilitate efforts to build an extensive resource of metabolic and cellular functions, including tasks unique to individual organisms. Such exhaustive lists of tissue- and/or organism-specific metabolic features can be developed and validated, as we did, on the basis of existing knowledge from the literature. However, further experimental validation will be important to more objectively benchmark the new tasks.

In this context, a major value of this work will be to propose cell-type- or tissue-specific functions based on transcriptomic data. To facilitate further validation of predicted tissue-specific task beyond established literature observations, one could use various databases. For example, we tested whether ontological information available in the Human Metabolome Database (HMDB) (Wishart et al., 2018) could cross-validate the tissue-specific human metabolic functions identified on the basis of the Human Protein Atlas dataset. Sixty-four of our metabolic tasks can be translated into the accumulation of a metabolite of interest listed in HMDB for which ontological data are available. We found that 73.2% of the tissue specificities listed in HMDB for these metabolite accumulations corroborated with identified tissue-specific metabolic tasks (Table S2). The increasing availability of other public experimental data, through consortia such as Human Cell Atlas (www.humancellatlas.org), EcoCyc (www.ecocyc.org), and Saccharomyces Genome Database (www.yeastgenome.org), will definitively facilitate such validation while also enabling the curation of new metabolic tasks for various model organisms.

The inclusion of other biological processes (e.g., transcription, translation) can easily be formulated into our framework by using different types of models (Thiele et al., 2012; Lerman et al., 2012), as our approach only requires gene information. Furthermore, gene ontology repositories could provide a starting point to identify new tasks by mapping existing gene sets onto genome-scale metabolic networks. Finally, future work will investigate contributions from different isoenzymes within each metabolic task, given that different cells and tissues can present the same metabolic reactions but using different isoenzymes with different activities (Uhlén et al., 2015). This variation in enzyme usage might underlie adaptations of metabolism to biological perturbation such as a disease. The CellFie framework can be further used to study other omics data, including proteomics, assay for transposase-accessible chromatin using sequencing (ATAC-seq), and any other type that can quantify genes or proteins. For example, in proteomics, one will input abundance of proteins associated to each reaction involved in a metabolic task instead of selecting the gene that will be the main determinant of gene abundance. Also, this could be used to uncover previously unknown protein functions or inversely to associate a new metabolic function with prior knowledge at the level of the protein. In this context, we anticipate that co-expression analysis and studies of protein structures will complement biochemical assays to assign activities to new proteins, which can then be added to the genome-scale models and existing or new metabolic tasks.

In conclusion, this framework provides an approach to contextualize gene expression data. Combined with knowledge-based functional analysis, this might, one day, enable the complete description of the molecular basis of any biological system based on a simple omics data analysis.

### Limitations of the study

The list of metabolic tasks in this study represents a limited collection of curated metabolic functions in human cells. The aim of this study was not to create and benchmark all metabolic tasks but rather to standardize previously identified metabolic tasks and use these to develop a tool for data analysis. Although the curated list covers a substantial proportion of human metabolic genes, there is a need for further work to describe the metabolic tasks involving the remaining genes not covered in the current task list, including those tasks unique to individual non-human organisms.

The list should also be expanded for other mammalian cells and extended to diverse organisms, along with more cellular functions captured in systems biology models of metabolism, transcription, translation, and signaling. However, further experimental validation will be important to objectively benchmark these new tasks, given that to date there is no exhaustive list of cell-, tissue-, and/or organism-specific metabolic functions. In this context, a major value of this work will be to propose cell-type- or tissue-specific functions based on transcriptomic data.

The presented framework currently only relies on the usage of transcriptomic data. The method could be adapted to study other omics data, including proteomics, ATAC-seq, and any other type that can quantify genes or proteins. Such applications would be beneficial to uncovering previously unknown protein functions or inversely associated new metabolic functions thanks to prior knowledge at the level of the protein.

## STAR★Methods

### Key resource table

**Table:** 

Reagent or resource	Source	Identifier
**Deposited data**		

RNA-Seq data for the 32 human tissues	Uhlén et al., 2015	proteinatlas.org
Adult mouse brain single-cell transcriptomic dataset	Saunders et al., 2018	dropviz.org
Human brain single-cell transcriptomic dataset from ROSMAP (Religious Orders Study and Memory Aging Project).	Bennett et al., 2018	radc.rush.edu

**Software and algorithms**		

GenePattern	Reich et al., 2006	genepattern.org
Cobra Toolbox 3.0	Heirendt et al., 2019	github.com/opencobra/cobratoolbox
Escher	King et al., 2015	escher.github.io

**Other**		

iMM1415	Sigurdsson et al., 2010	bigg.ucsd.edu
Recon2.2	Swainston et al., 2016	bigg.ucsd.edu

### Resource availability

#### Lead contact

Further information and requests for resources and reagents should be directed to and will be fulfilled by the lead contact, Dr. N.E. Lewis (nlewisres@ucsd.edu).

#### Material availability

This study did not generate new unique reagents.

#### Data and code availability

The code sources to compute the metabolic task score are available as a MATLAB package at https://github.com/LewisLabUCSD/CellFie and as a module of GenePattern at www.genepattern.org.

### Methods details

#### Curation of metabolic tasks

The curation was done by first taking the union of previously published lists of metabolic tasks (Blais et al., 2017; Thiele et al., 2013). We removed duplicated tasks and lumped tasks that rely on the description of similar metabolic functions. Each remaining task without strong biological evidence was removed. We also created 9 new tasks that were essential for the acquisition of already described metabolic functions (i.e., intermediate biosynthetic steps for the acquisition of other tasks). Doing so, we obtained a collection of 195 tasks associated with 7 systems (energy, nucleotide, carbohydrates, amino acid, lipid, vitamin & cofactor and glycan metabolism). For each task, we provided its original source (Recon and/or iHsa) and comments on the biological evidence of this metabolic function (Table S1).

#### Inference of metabolic tasks from transcriptomic data

The “metabolic tasks” framework has been previously used to evaluate the quality and capabilities of genome-scale metabolic models in multiple publications (Duarte et al., 2007; Thiele et al., 2013; Blais et al., 2017; Gille et al., 2010; Mardinoglu et al., 2014; Uhlén et al., 2015; Agren et al., 2014; Bordbar et al., 2012). We recently unified the formalism of the metabolic tasks and the associated computational framework for their use in the modeling context (details are presented in our earlier study (Richelle et al., 2019a)) but also benchmarked the methods used to process gene expression data for such computational analysis (Richelle et al., 2019b).

The metabolic task framework presented in Richelle et al., 2019ahad to be adapted to enable the direct inference of metabolic task scores from the transcriptomic data, and in doing so, extend the application of the concept beyond the model benchmarking scope. To this end, we extracted the reaction sets associated with each metabolic task and accessed to the list of genes that may contribute to the acquisition of this metabolic function based on the GPR rules. Specifically, we used the Parsimonious Flux Balance Analysis (pFBA) to define the smallest set of reactions and associated genes required to pass a task within a specified model (Lewis et al., 2010a). The way metabolic task has been defined (i.e., capacity of producing a defined amount of an output products set when only a defined list of input substrates is available in defined quantities) ensures that only the shortest metabolic route can be used to perform a task, which is a valid statement for the proposed list of tasks. Thanks to the availability of this information, metabolic functions can now be directly assessed from transcriptomic data.

Specifically, the computation of metabolic task scores relies first on the definition of the set of active genes in each cell or tissue. As presented in our benchmarking study (Richelle et al., 2019b), there are many different ways to perform this preprocessing step. Therefore, all the results presented in the present publication have been computed by using the preprocessing parameter combination presenting the best performance (i.e., combination “*Local T2* + *GM1* + *Order 2*”). In brief, a local thresholding approach using lower and upper bounds on the gene activity profile (i.e., respectively, the 25^th^ and the 75^th^ percentile of the overall gene expression value distribution) is implemented to attribute a score to each gene.

$$Gene\;Score=5\cdot \log\left(1+\frac{Expression\;level}{Threshold}\right)$$

These gene scores are further mapped to the genome-scale model by parsing the GPR rules (i.e., selection of the *minimum* expression value amongst all the genes associated to an enzyme complex -AND rule- and the *maximum* expression value amongst all the genes associated to an isozyme -OR rule (Jensen et al., 2011)) associated with the set of reactions representing one metabolic task. Therefore, each reaction involved in a task is associated with a reaction activity level (RAL) that corresponds to the preprocessed gene expression value of the gene selected as the main determinant for this reaction.

We also computed the significance of each gene selected with regard to its overall use throughout the whole metabolism in the observed condition. Some genes will be mapped to multiple reactions (e.g. promiscuous enzyme). Therefore, we assume that there may be some competition between the reactions using this gene. We define the significance of a gene (S) by its specificity for a reaction. It is computed as the inverse of the number of reactions in which this gene is used as the main determinant. Finally, the metabolic score can be computed as the mean of the product of the activity level of each reaction with the significance of its associated gene:

MTscore=sum(RAL∗S)/numberofreactionsinvolvedinthetask

MT score values enable the relative quantification of the activity of a metabolic task in a specific condition based on the availability of data for multiple conditions. Indeed, some important housekeeping genes always present at very low expression values. Therefore, a metabolic function that will completely rely on this set of genes will always result in a low MT score. Contrarily, some tasks can be associated with highly expressed genes. Therefore, MT scores cannot be compared across tasks but only across samples. To partly overcome this problem, we also propose this scoring approach in its binary version to determine whether a metabolic task is active or not based on a gene expression profile. To this end, a metabolic task will be considered as active if the average of its associated RAL is superior to 5log(2).

#### Assessment of tissue similarities

We computed the scores of the 195 metabolic tasks in their continuous version based on the transcriptomic data available for 32 different tissues in the Human Protein Atlas (Uhlén et al., 2015) dataset using Recon2.2 (Swainston et al., 2016) as the reference genome-scale metabolic model (Swainston et al., 2016). These scores were used to compute the Euclidean distance between each tissue. We associated each tissue to an organ system as defined in the Human Protein Atlas (Uhlén et al., 2015) (Table S3) and computed the average Euclidean distance between tissues belonging to the same organ system. Note that, we only considered organ systems presenting more than two tissues within the same group (i.e. Female Reproductive, Lymphatic and Gastrointestinal – total of 15 tissues). To compute the significance of our results, we generated the mean Euclidean distance for 10000 randomly selected groups with the same number of tissues (i.e. random selection of 3 tissues among the 15 considered for the Female Reproductive group) and computed the exact p value (i.e. proportion of random distance lower than the observed distance) associated to each organ system. We also performed this analysis using the metabolic scores when computed in their binary version (Figure S1 and Table S2). The histological information used in the assessment of tissue similarities has been collected from the microscopy images and associated description available in the Human Protein Atlas (Uhlén et al., 2015).

### Quantification and statistical analysis

#### Principal component analysis for differentiating brain cell-types

A matrix representing the metabolic function scores for 3 brain cell types (i.e., astrocytes, neurons and oligodendrocytes) was constructed by multiplying the metabolic task scores computed in their continuous version (Table S4) with the ones in their binary version (Table S5). A PCA analysis on this matrix was conducted. As this analysis did not enable the differentiation between astrocytes and oligodendrocytes, we performed a subsequent similar PCA analysis by only using the samples related to these specific cell-types.

#### Clustering of excitatory neurons samples from the ROSMAP dataset

We clustered the samples identified as excitatory neurons by identifying the tasks that were active in more than 50% of the dataset. This threshold has been set with respect to the percentage of excitatory neurons samples associated with a positive diagnosis of Alzheimer’s disease (i.e., 51,2%). Only three metabolic tasks correspond to this criterion: the conversion of phosphatidyl-1D-myo-inositol to 1D-myo-inositol 1-phosphate, the synthesis of tetrahydrofolate synthesis and the synthesis of Tn antigen (Glycoprotein N-acetyl-D-galactosamine. We further used them to divide the samples into 8 metabolic clusters depending on the combination of their activity in each sample (Figures 6A and 6B). Note that prior to this choice, other clustering methods have been investigated. Our first approach was using k-means clustering. To this end, we used the percentage of coordinates that differ (hamming distance) in the binary matrix of the metabolic task score (active vs non-active) and the matlab function k-means with 10 replicates. To identify the appropriate number of clusters to separate the data, we computed the within-cluster sum of square distance (wws) and the average silhouette value by iteratively increasing the number of clusters from 1 to 15. This approach also led to the identification of 8 metabolic clusters that were displaying the same metabolic dysregulations. In order to ensure the reproducibility of the results presented, we preferred to use a more straightforward clustering method.

We compared the metabolic clusters obtained with our approach to the clusters identified in a publication (Mathys et al., 2019) using the ROSMAP data (Figure S5). We can observe that the metabolic clusters M3 and M4 are only enriched in clusters Ex2 and Ex4 who were identified as highly correlated with Alzheimer’s pathological traits in the reference publication. The same observation can be done with M6 metabolic cluster and Ex6, the cell type cluster identified as highly correlated with patients without Alzheimer’s disease.

### Additional resources

#### Analysis with the GenePattern CellFie module

We created a web-based CellFie module that has been integrated into the list of tools available in GenePattern (Reich et al., 2006) (www.genepattern.org). A tutorial explaining how to run CellFie as a GenePattern module is available on the wiki section of the github repository: https://github.com/LewisLabUCSD/CellFie. This repository includes the source code of the computational framework running on Matlab. The source code has been developed based on functions from the Cobra Toolbox (Heirendt et al., 2019). It also includes a tutorial to visualize the output results of CellFie on metabolic maps using Escher (King et al., 2015). The metabolic task score can be computed based on any type of transcriptomic data type (e.g., microarray or RNA-seq, bulk or single cell) regardless of data unit as long as the whole dataset has been generated from the same analytical platform. CellFie can also be used to compute metabolic tasks for CHO cells, rat and mouse.

## Supplemental information

Document S1. Figures S1–S5

Table S1. Exhaustive description of the curated list of metabolic tasks, related to Figure 1

Table S2. Binary and continuous versions of the metabolic task scores of 32 tissues from Human Protein Atlas and cross-validation of information with Human Metabolome Database, related to Figure 2

Table S3. Association between tissue and organ system as listed in Human Protein Atlas, related to Figure 2

Table S4. Continuous version of the metabolic task scores of 458 single brain cells of adult mouse from Single-Cell Atlas of Adult Mouse Brain, related to Figures 4B and 5

Table S5. Binary version of the metabolic task scores of 458 single brain cells of adult mouse from Single-Cell Atlas of Adult Mouse Brain, related to Figures 4B and 5

Table S6. List of tasks associated with neurotransmitter synthesis, related to Figure 5D

Table S7. Repartition of the samples from ROSMAP dataset among each identified metabolic subcluster for each patient, related to Figure 6

Document S2. Article plus supplemental information
