# Supplementary material for: Model-based assessment of mammalian cell metabolic functionalities using omics data
Source: Cell Rep Methods. 2021 Jun 30;1(3):100040. doi: 10.1016/j.crmeth.2021.100040 (PMC8577426; doi:10.1016/j.crmeth.2021.100040)
Supplement: Document S1. Figures S1–S5 [file mmc1.pdf]

Cell Reports Methods, Volume *1*

Supplemental information

Model-based assessment of mammalian cell metabolic functionalities using omics data

Anne Richelle, Benjamin P. Kellman, Alexander T. Wenzel, Austin W.T. Chiang, Tyler Reagan, Jahir M. Gutierrez, Chintan Joshi, Shangzhong Li, Joanne K. Liu, Helen Masson, Jooyong Lee, Zerong Li, Laurent Heirendt, Christophe Trefois, Edwin F. Juarez, Tyler Bath, David Borland, Jill P. Mesirov, Kimberly Robasky, and Nathan E. Lewis

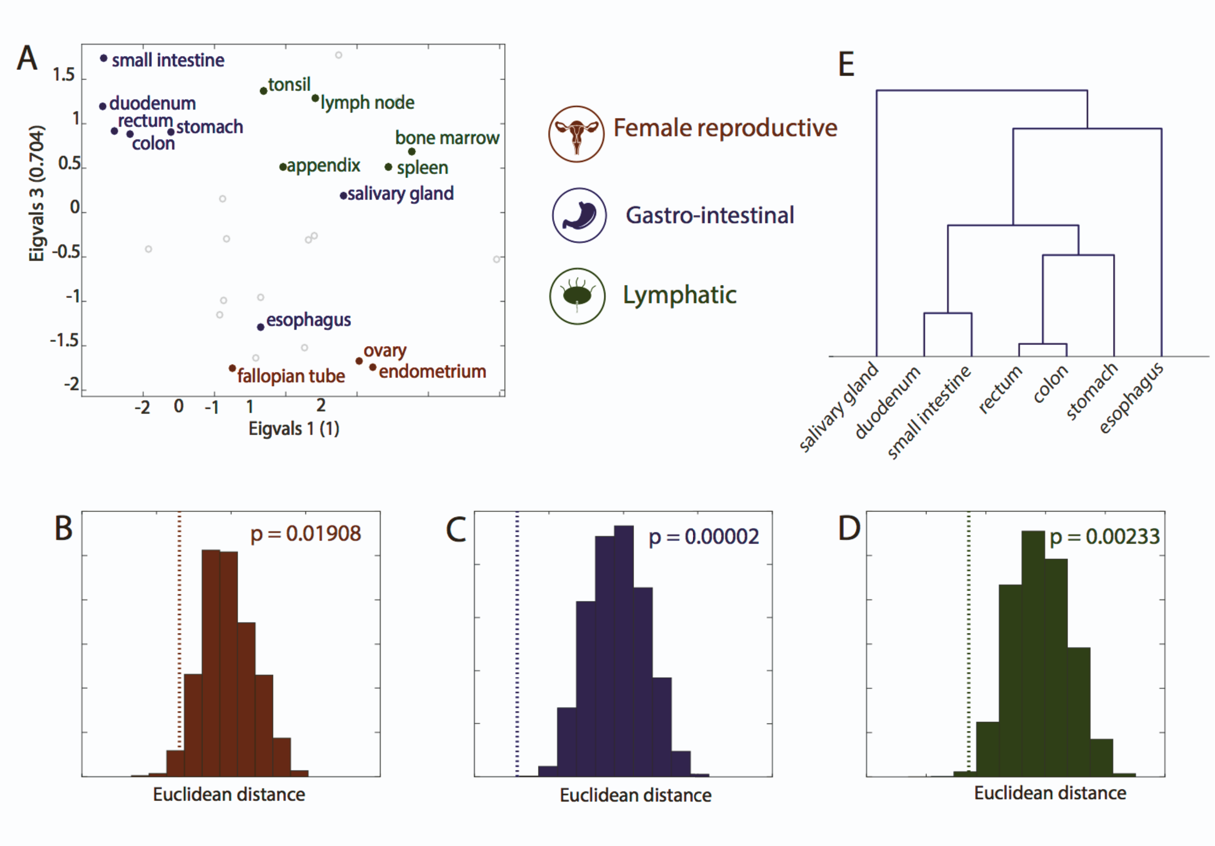

Figure S1, Related to Figure 3. **Metabolic task in its binary version still better captures tissue groups at organ system level -** (A) Visual representation of the similarity between tissues using a principal coordinates analysis (B, C & D). Significance of tissue grouping at the organ system level. Distribution of mean Euclidean distance for 10000 randomly selected group with the same number of tissues. The dotted line is the mean Euclidean distance between tissues belonging to the same organ system and its associated p-value (see Methods for more details). (E) Hierarchical clustering of similarities between tissues of the gastrointestinal group.

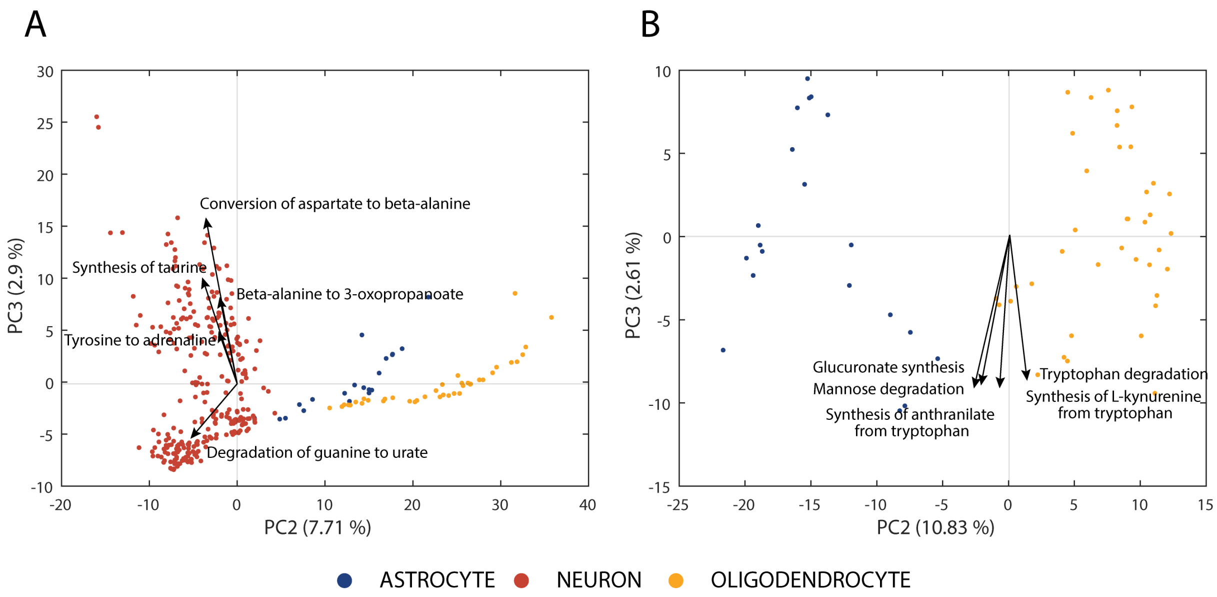

Figure S2, Related to Figure 5. Principal Components Analysis of the metabolic differences between astrocytes, neurons and oligodendrocytes - (A) Component scores plot of the PCA performed using the 3 different cell types (astrocytes, neurons, oligodendrocytes) and the 5 tasks influencing the most the third principal component. (B) Component scores plot of the PCA performed using only 2 cell types (astrocytes, oligodendrocytes) and the 5 tasks influencing the most the third principal component.

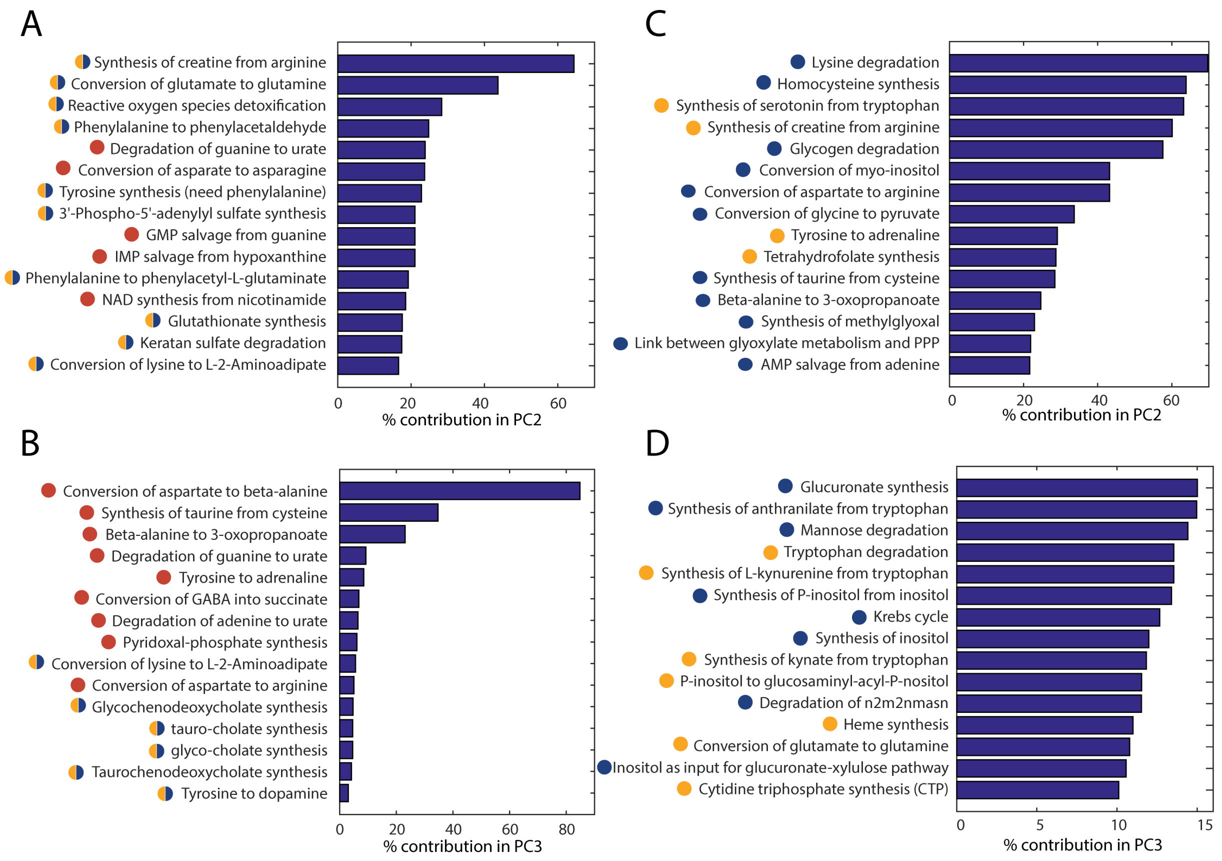

Figure S3, Related to Figure 5. PCA loadings of the metabolic differences between astrocytes, neurons and oligodendrocytes - Contribution of the task in the principal components 2 (A) and 3 (B) in the analysis performed using the three different cell types (astrocytes, neurons, oligodendrocytes) and in the principal components 2 (C) and 3 (D) in the analysis performed using the samples associated to astrocytes and oligodendrocytes. Note that we did not plot the contribution of variables in PC1 has this component was not informative to differentiate the cell-types. The color bullets represent the direction of the loadings for the separation between the different cell-types (blue = astrocytes, yellow = oligodendrocytes and red = neurons).

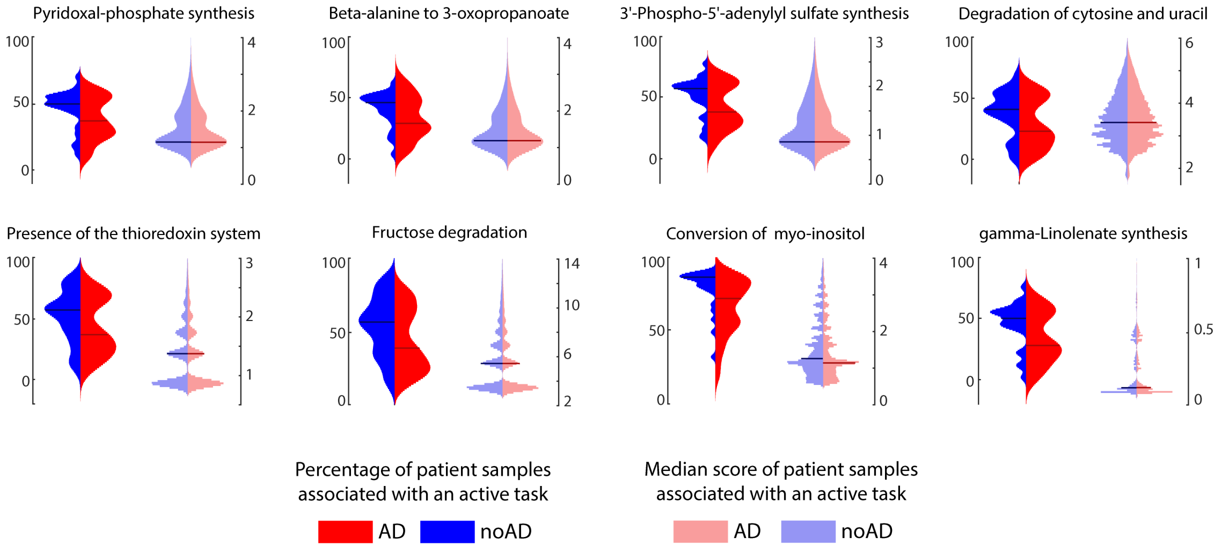

Figure S4, Related to Figure 6. Expression pattern of the metabolic tasks presenting a dysregulated activity across a group of patients with different diagnosis for Alzheimer's disease - Left pattern - percentage of patient samples associated with an active task and right pattern - related median score. Blue part of the pattern– patients without Alzheimer and red part of the pattern – patients with Alzheimer). The horizontal lines represent the median of the distribution.

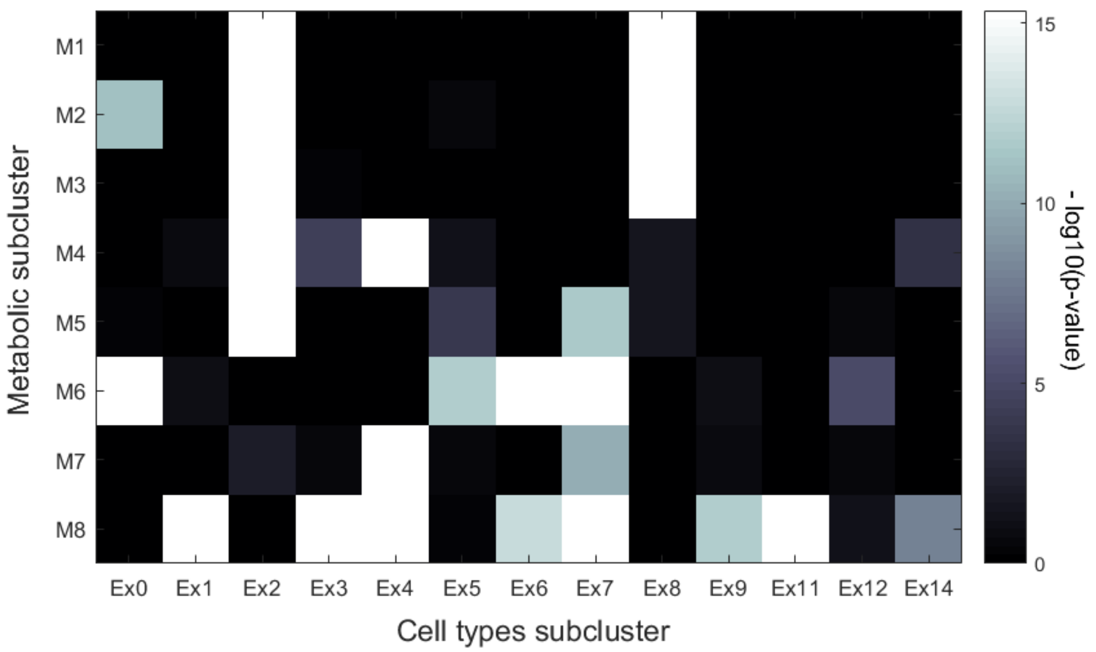

Figure S5, Related to STAR Methods. Comparison of the metabolic clusters identified using the ROSMAP data in Mathys *et al.* (2019) with the ones presented in this study - Enrichment analysis (hypergeometric test) within the cell types subclusters identified in the original reference paper (columns) of the metabolic subclusters identified with our approach (rows).
